# Causal mutations from adaptive laboratory evolution are outlined by multiple scales of genome annotations and condition-specificity

**DOI:** 10.1186/s12864-020-06920-4

**Published:** 2020-07-25

**Authors:** Patrick V. Phaneuf, James T. Yurkovich, David Heckmann, Muyao Wu, Troy E. Sandberg, Zachary A. King, Justin Tan, Bernhard O. Palsson, Adam M. Feist

**Affiliations:** 1 Bioinformatics and Systems Biology Program, University of California, San Diego, La Jolla, CA 92093 USA; 2 Institute for Systems Biology, Seattle, WA 98109 USA; 3 Department of Bioengineering, University of California, San Diego, La Jolla, CA 92093 USA; 4 Department of Pediatrics, University of California, San Diego, 9500 Gilman Drive, La Jolla, CA 92093 USA; 5 Novo Nordisk Foundation Center for Biosustainability, Technical University of Denmark, Building 220, Kemitorvet, 2800 Kgs. Lyngby, Denmark

**Keywords:** Adaptive laboratory evolution, Mutation functional analysis, Mutation meta-analysis, Mutation convergence, Multiscale genome annotation

## Abstract

**Background:**

Adaptive Laboratory Evolution (ALE) has emerged as an experimental approach to discover mutations that confer phenotypic functions of interest. However, the task of finding and understanding all beneficial mutations of an ALE experiment remains an open challenge for the field. To provide for better results than traditional methods of ALE mutation analysis, this work applied enrichment methods to mutations described by a multiscale annotation framework and a consolidated set of ALE experiment conditions. A total of 25,321 unique genome annotations from various sources were leveraged to describe multiple scales of mutated features in a set of 35 *Escherichia coli* based ALE experiments. These experiments totalled 208 independent evolutions and 2641 mutations. Additionally, mutated features were statistically associated across a total of 43 unique experimental conditions to aid in deconvoluting mutation selection pressures.

**Results:**

Identifying potentially beneficial, or key, mutations was enhanced by seeking coding and non-coding genome features significantly enriched by mutations across multiple ALE replicates and scales of genome annotations. The median proportion of ALE experiment key mutations increased from 62%, with only small coding and non-coding features, to 71% with larger aggregate features. Understanding key mutations was enhanced by considering the functions of broader annotation types and the significantly associated conditions for key mutated features. The approaches developed here were used to find and characterize novel key mutations in two ALE experiments: one previously unpublished with *Escherichia coli* grown on glycerol as a carbon source and one previously published with *Escherichia coli* tolerized to high concentrations of L-serine.

**Conclusions:**

The emergent adaptive strategies represented by sets of ALE mutations became more clear upon observing the aggregation of mutated features across small to large scale genome annotations. The clarification of mutation selection pressures among the many experimental conditions also helped bring these strategies to light. This work demonstrates how multiscale genome annotation frameworks and data-driven methods can help better characterize ALE mutations, and thus help elucidate the genotype-to-phenotype relationship of the studied organism**.**

## Background

Adaptive Laboratory Evolution (ALE) is used to study microbial populations under specific conditions over many generations and provides insights into the underlying mechanisms of adaptive phenotypes. Mutations observed from ALE experiments have proven valuable for both biological discovery and applied biotechnology, such as the elucidation of the rate and mechanisms of mutation development [1–5] and the design of industrially relevant strains for increased bioproduction [6]. The increased scale of ALE experiments—due to the low cost of sequencing and the inclusion of intermediate/midpoint samples [7], multiple replicates [8], and population samples [9]—has increased the number of mutations that require analysis. While the identification of “*key mutations*”, or those mutations hypothesized as being adaptive, is better enabled with more mutation data [8], traditional methods are not well suited for large scale sets of ALE mutations. The potential of mutated non-coding regulatory features contributing to an adaptive phenotype further complicates the set of features to consider when seeking to understand ALE mutations. Moreover, multiple experimental parameters can contribute to the selection pressure that an organism experiences in experimental evolution [6, 10]. Ultimately, the primary challenges with traditional mutation functional analysis are finding the subset of adaptive mutations among the many that emerge during an ALE experiment and understanding the adaptive mechanisms of these mutations relative to specific selection pressures.

The main concern with traditional ALE mutation analysis is the mutated genes and how the sequence changes affect their function. Identifying commonly mutated genetic features (genes or intergenic regions) across replicate ALEs, known as convergence, has been established as a primary method for identifying potentially causal mutations, or key mutations, in ALE experiments [8]. Mutation convergence on broader levels of genomic organization has provided evidence that mutations targeting different features can accomplish similar adaptive functional changes [11]. This bottom-up convergence of mutated features across multiple scales of annotations enables a top-down approach to understand large sets of mutations: researchers can consider the broader functional annotations emphasized by large sets of small mutated features before analyzing individual mutations. Enrichment methods have been developed to identify over-represented classes among large collections [12]. Thus, enrichment methods can leverage high-throughput genome-wide data and molecular biology ontologies to identify enriched biological functions from large sets of mutated genes. However, the challenges of examining non-coding regulatory features and deconvoluting selection pressures for ALE mutations remain.

The accumulating wealth of information on the molecular biology of *Escherichia coli* K-12 MG1655 has led to the emergence of knowledge and data resources that can help solve challenges in understanding ALE results. Genome annotation frameworks such as regulons [13], pathways [14], and clusters of orthologous groups (COG) [15] describe functionally related coding and non-coding regulatory features on multiple scales of genome annotation. Similar to gene set enrichment analysis [12], significant enrichment can be investigated across multiple scales of genome annotations for meaningful convergence events. Additionally, the increased amount and scale of ALE experiments have led to efforts in consolidating their results. ALEdb, a web-based platform, reports on the mutations and experimental conditions from multiple experimental evolutions [16]. The mutations and conditions found in ALEdb can be used to associate mutated features to conditions and provide evidence on the selection pressures for ALE mutations.

Here, we address the challenges with finding and understanding adaptive mutations through two approaches. The first is to better identify key mutations than traditional means by seeking statistically significant mutation convergence across multiple scales of genome annotations. The second is to better understand these key mutations through their enrichment of functional annotations and their statistical associations to experimental conditions. We anticipate that the approaches described here will provide the ability to deconvolute systematic targets of adaptive mutations and their selection pressures to aid in improving ALE mutation functional analysis.

## Results

### A framework for finding key mutations using significant convergence on multiple scales of genome annotations

**Fig. 1:**
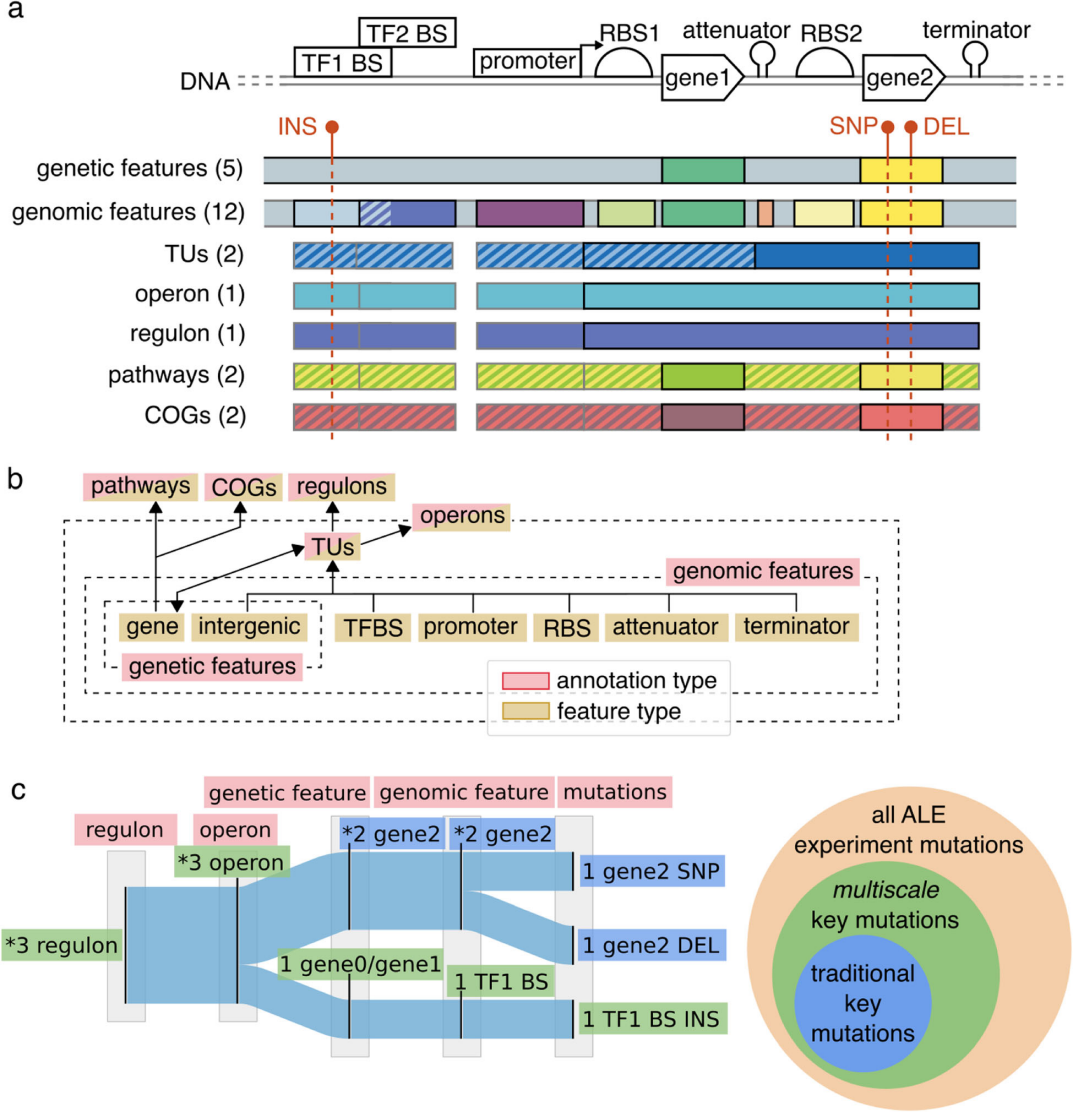
An illustration of how features were connected together in this work’s multiscale annotation framework. **a** An illustration of the variety of annotations, given in parenthesis per annotation type, for the *E. coli* K-12 MG1655 genome and how a single mutation can affect multiple features across different scales of annotations. The diagonal striped regions illustrate overlapping features. Dark-edged rectangles represent defining features for an annotation type. Grey-edged boxes represent operational regions or features associated with the defining features. Features are not to scale. (**b**) A flow diagram demonstrating the mapping of mutated small-scale features onto larger-scale features of the multiscale annotation framework of *E. coli* K-12 MG1655 from this work. **c** An example of the Sankey diagram visualization used in this work to demonstrate the number of mutations to each feature and the connectivity of smaller-scale features to their larger-scale counterparts. Each feature is annotated with a value representing the number of instances the feature was observed to be mutated. Significantly enriched features are annotated with an asterisk (*). Mutated features contributing to the significant enrichment of higher-level annotations are considered as hosting key mutations. The venn diagram illustrates the potential for finding more key mutations than traditional methods through multiscale scale annotation mutation convergence

**Fig. 2:**
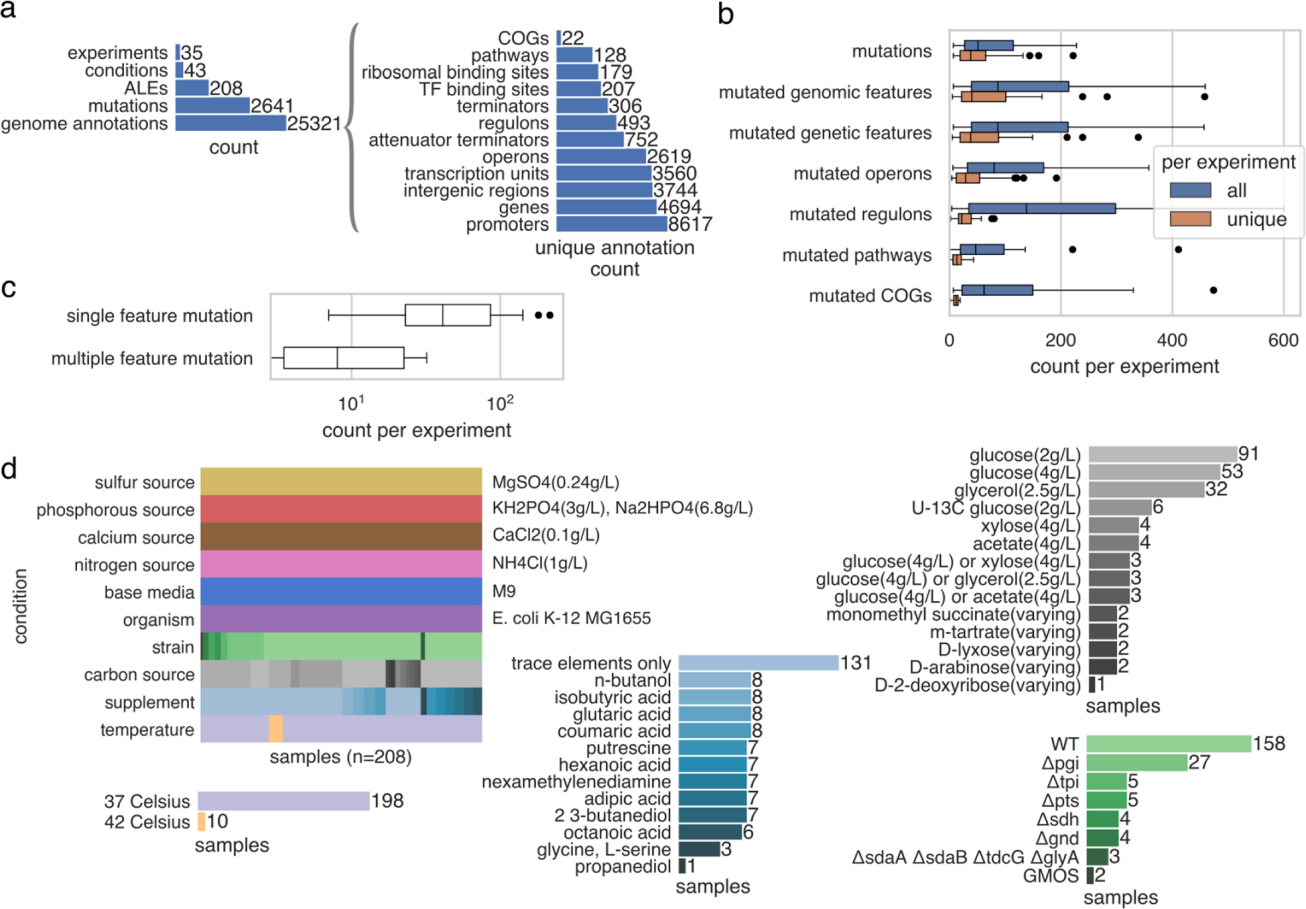
Magnitudes for different aspects of the ALE experiment mutations, conditions, and annotations used within this study. **a** The total number of experiments, ALEs, mutations, conditions, and unique genome annotations. **b** The distribution of mutations and annotation type features mutated per experiment. **c** The distribution of single versus multiple feature mutations per experiment, based on genomic features. **d** The conditions describing ALE experiments from ALEdb, the combinations of condition labels across ALEs, and the amount of each specific label for conditions with multiple labels

To comprehensively characterize the frequency of mutated features on the genome for a set of ALE experiments, mutation annotations should consider the variety of non-coding regulatory features along with coding features. Genome references typically include gene annotations, enabling mutation calling pipelines to describe mutations as affecting genes and/or intergenic regions, though there exists a multitude of additional feature and functional annotations. In this work, multiple annotation types were used to consider different scales of mutated features (i.e., gene, operon, regulon, etc.) for the *E. coli* K-12 MG1655 genome (Fig. 1a), with a total of 25,321 unique genome annotations for mutations (Fig. 2a). The smallest-scale genome annotation type used in this work was referred to as genomic features and described coding features, non-coding regulatory features, and non-coding intergenic regions of unknown function. The regulatory features considered were transcription factor binding sites (TFBS), promoters, terminators, attenuator-terminators, and ribosome binding sites (RBS). These small regulatory features and genes were described by RegulonDB version 10.0 [17]. Mutated coding and non-coding features were then mapped to their transcription units (TU), then operons and functional annotations (Fig. 1b). The sources of functional annotations used in this work were pathways as described by the PATRIC database [14], regulons of RegulonDB version 10.0 [17], and clusters of orthologous groups (COGs) [15]. This multiscale annotation framework included a level of annotation with only genes and intergenic regions to provide the expected evidence of convergence according to previously established methods of finding key mutations [8].

The Sankey diagrams [18] visualizations used in this work demonstrate the number of mutations to each feature and the connectivity of smaller-scale features to their larger-scale counterparts (Fig. 1c). The underlying data structure is a directed acyclic graph (DAG). The DAG represents the frequency of mutated genomic features and how they aggregate towards broader annotation type features. The nodes in the DAG represent a mutated feature. The edges of the DAG represent the connection between those features, where their direction typically goes from smaller to broader annotation types (Fig. 1b). The edge weights represent the accumulating instances of mutated genomic features across annotation scales. The node weights represent the sum of incoming edge weights. The visualization presented in this study for the DAG additionally includes the different mutation types affecting each genomic feature. A DAG is constructed per mutation set, where the mutated genomic features are first established from the mutation information. A single mutation may introduce multiple genomic features as well as multiple mutations may only contribute to the mutation frequency of one genomic feature. If no explicit genomic feature can be connected to a mutation, an intergenic region annotation is assigned according to flanking genes. Transcription units (TU) are part of the DAG, though aren’t included in this study’s Sankey diagrams due to containing mostly redundant mutated feature convergence with operons. TUs connected genes to higher-level annotations associated with regulatory mechanisms. TUs also enabled the connection between non-coding features and gene-based functional annotations, such as pathways and COGs, according to the genes hosted on a TU (Fig. 1b). Connections between mutated genomic features and broader annotations mostly rely on the relationships established within the multiple sources of operational and functional annotations integrated for this study. Mutated intergenic regions of unknown function are assigned TUs according to overlapping nucleotide positions. After TUs are assigned, functional annotations can be connected in the same manner as with mutated genomic features of known functions.

Large features are aggregations of many smaller features and consequently manifest mutation convergence more easily by random chance (Fig. 2b). A statistical enrichment method was applied to quantify the significance of mutation convergence on features of the genome and prioritize their importance for functional analysis. The method assumed that each nucleotide in the genome has the same probability of being spontaneously mutated in an ALE. This assumption translated to annotated features on the genome having a probability of being mutated proportional to their length. Though this assumption may not perfectly reflect the distribution of mutations across a genome, it has been experimentally validated to represent their general distribution with mutation accumulation studies [18]. Thus, studies searching for signals of mutation selection commonly use a random distribution of mutations across the amino acids or nucleotides for a set of features [19] or a whole genome [20] as a null hypothesis in their statistical enrichment tests. Significant enrichment of annotated features was tested separately per annotation type (genomic features, genetic features, operons, etc). For annotation types that don’t have explicit coverage of the entire genome, an additional feature was added to the annotation type set that represented these non-annotated regions. For each annotation type, a permutation test of 10,000 iterations was executed where mutations were distributed across a specific annotation type’s features using their mutation probabilities per iteration [19]. The length of each regulon was defined as the total number of nucleotides in the TUs considered within each regulon. The length of each COG or pathway was defined as the total number of nucleotides in the TUs in which the gene connected to the COG or pathway was found, excluding the lengths of genes within the TU not connected to the specific COG or pathway. Features with more than one mutation and a permutation test *p*-value < 0.05 (Bonferroni corrected) were considered to be significantly enriched by mutations. A mutated feature found to be significantly enriched had an asterisk (*) prepended to its label within the flow diagram (Fig. 1c). Finally, all mutations contributing through convergence to the significance of an enriched feature were considered key mutations (Fig. 1c).

### The amount and diversity of ALE mutated feature types and experiment conditions

The dataset used within this work contained 35 *Escherichia coli* K-12 MG1655 based ALE experiments from ALEdb [16], totaling 208 independent evolutions and 2641 mutations (Fig. 2a). Within this dataset, experiments have a median of 51 mutations, with a median of 38 being unique (Fig. 2b). As broader annotations types are considered, a smaller amount of unique features are mutated per ALE experiment (Fig. 2b). Multi-nucleotide structural variants or overlapping features (Fig. 1a) on the genome can result in more than one genomic feature affected by a mutation (Fig. 2c), especially in the case of the numerous small regulatory features (Fig. 2a), therefore leading to more mutated features than mutations in an ALE experiment. Some of these small non-coding features can additionally regulate more than one operon, further complicating the analysis of their effects on the host. These types of mutations and relationships between features increase the number of artifacts to consider with the functional analysis of ALE mutations. In this dataset, mutations affected a median of 87 genomic features per experiment (Fig. 2b), demonstrating that there could be more mutated features in an ALE experiment than individual mutation events.

This dataset tracked 10 different types of experimental condition types with a total of 43 unique conditions (Fig. 2a, d), describing the organism and environment of the ALE experimental design. Annotations affected by mutations were statistically associated with these conditions to better understand which may have selected for the ALE mutations. Fisher’s Exact Test was used for the statistical association, where a condition and mutated feature were considered associated if their odds ratio was greater than 1 with *p*-value < 0.01 (Bonferroni corrected). A *p*-value < 0.01 was used to measure significance rather than a *p*-value < 0.05 to reduce the amount of false-positives with associations.

### Applying methods across a large consolidated ALE mutation dataset and to individual case studies

The impact of annotating mutations using multiscale genomic features was explored using 18 *E. coli* K-12 MG1655 based ALE experiments. We first present a small case study from a Δ*pgi* ALE experiment [21, 22] to demonstrate how the mutation of both coding and non-coding features can accomplish the same fitness benefit. Further, the abundance of both coding and non-coding features, along with their contributions to key mutation identification across the data set, was investigated. We then examined the impact of these methods on the number of key mutations found across these ALE experiments. Finally, we applied these methods to the mutation sets of two ALE experiments separately, to demonstrate the novel value of the new types of evidence with mutation functional analysis. The first ALE experiment represents a newly released mutation set from a previous ALE growth-rate characterization study to growth on glycerol as a carbon source [23]. The second ALE experiment represents a previously published mutation set from an L-serine tolerance ALE [24] with a newly outlined adaptive strategy based on evidence from this work’s methods.

#### Key mutations increase through the bottom-up convergence of mutated features on a multiscaled annotation framework

**Fig. 3:**
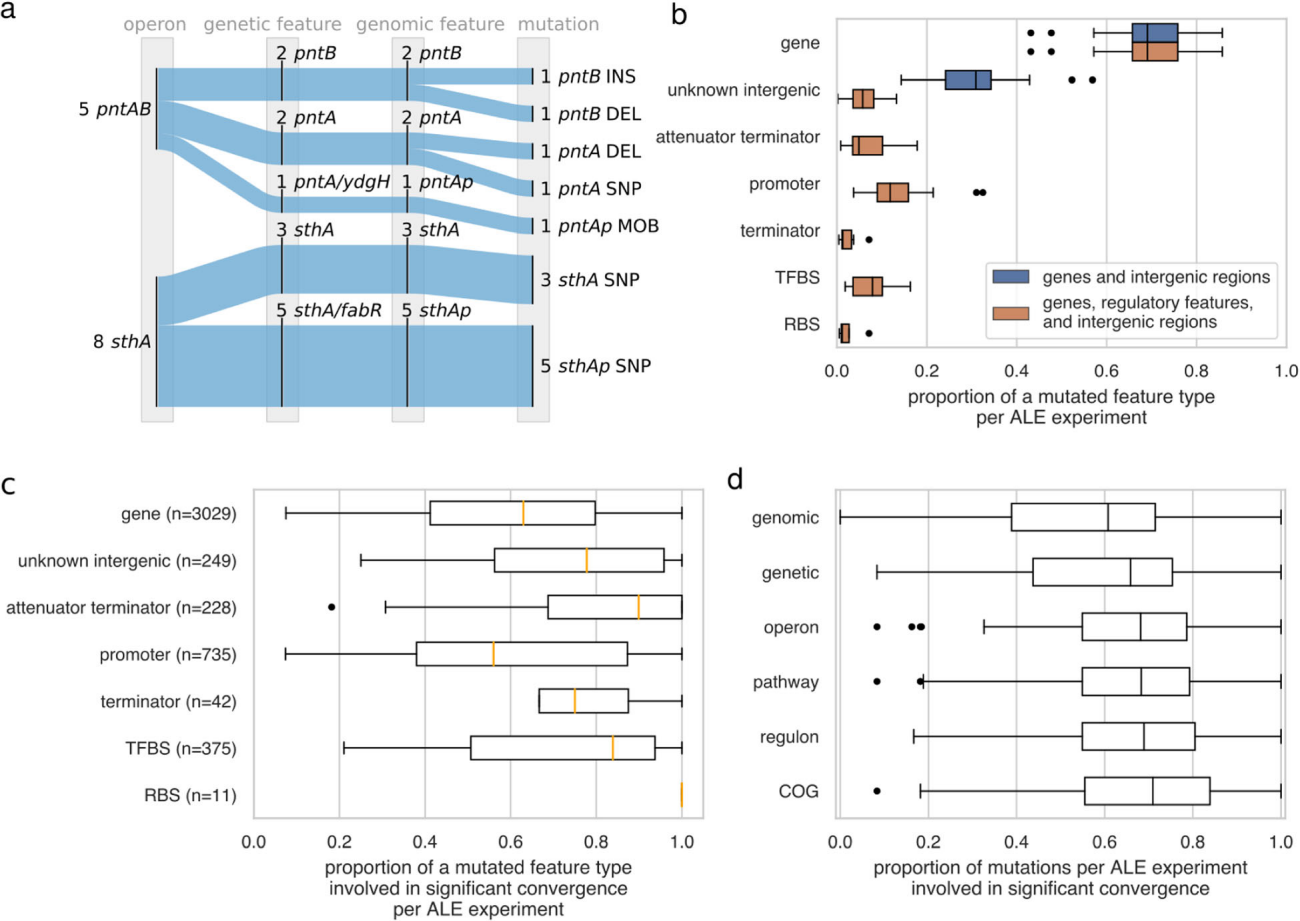
The amount of identified key mutations increase through the bottom-up convergence of mutated features on a multiscaled annotation framework. **a** The convergence of mutated genes and regulatory features onto *pntAB* and *sthA* operons from a *Δpgi* ALE experiment on *E. coli* K-12 MG1655 [21, 22]. **b** Proportions of mutated features according to different genomic feature types. The distributions were generated by finding the proportion of each mutated feature per ALE experiment. **c** The proportions of mutated feature types across ALE experiments that were involved in significant convergence (*P* < 0.05, permutation test, mutations to feature > 1) on the multiscale annotation framework. The number of times each feature type was observed mutated is included (‘n’). (**d**) Proportion of mutated features across ALE experiments that had significant convergence up to an annotation type on the multiscale annotation framework of Fig. 1

The convergence of mutated coding and non-coding regulatory features onto broader annotations was investigated with existing ALE experiment mutations. Non-coding mutations have previously been seen to play a beneficial role in ALE phenotypes [22, 25]. To illustrate the convergence of both coding and non-coding mutations onto a broader scale of annotation, a case study around operons involved in transhydrogenase activity is presented using data from one of the ALE experiments consolidated in this study’s data set. This particular ALE experiment reported on the adaptation to a *pgi* knockout (i.e., Δ*pgi*), finding that the proteins PntA and PntB were rendered non-functional through truncations [22]. The study additionally observed a mobile insertion element mutation upstream of the *pntA* gene in a replicate lacking *pntA* and *pntB* mutations. The same ALE study had seen SNPs to both *sthA* and its upstream regions. The mutations to these upstream regions were hypothesized to change the expression of the downstream genes in a way that benefited the Δ*pgi* host. Advanced phenotypic characterization of the endpoint strains with *pntA*, *pntB*, *sthA*, and upstream mutations revealed a convergence to equivalent intracellular states, demonstrating the same phenotype achieved by different mutations. The potential impact of the upstream mutations became more evident when integrating annotations for non-coding regulatory features and observing the convergence of all small mutated features onto broader annotations such as operons (Fig. 3a). The upstream mutations targeted promoters for operons of the mutated *pntA*, *pntB*, and *sthA* genes. The convergence of mutations onto the *pntAB* and *sthA* operons highlighted their importance in this adaptation and serve to contextualize each mutation involved.

The abundance of regulatory features with key mutations was investigated with a set of *E. coli* K-12 MG1655 ALE experiments. A variety of non-coding regulatory features alongside genes was integrated to reduce the proportion of non-coding mutation targets with unknown function (Fig. 3b). It was observed that all small non-coding regulatory features had mutations involved in statistically significant convergence (*P* < 0.05, permutation test, mutations to feature > 1). Except for promoters, the median proportions of non-coding features involved in significant convergence across ALE experiments were higher than that of coding features (Fig. 3c). In fact, in all cases, mutated RBS were always seen to be involved in statistically significant convergence.

The convergence of small mutated features onto broader annotations and functions may not always be straightforward to manually identify with traditional methods. For example, an L-serine tolerance ALE experiment acquired adaptive mutations to *rho* or the *trxA/rhoL* intergenic region in different independent replicates [24]. The intergenic mutation targeted the *rhoLp* promoter for the operon hosting both *rhoL* and *rho* genes, demonstrating that beneficial mutations may be found in features not immediately adjacent to a given gene. Further, beneficially mutated features belonging to the same system are not always found on the same operon. For example, in an ALE experiment that resulted in a strain which had a elevated levels of aromatic amino acids, mutations to the *rcsA, rcsB*, and *yrfF* genes were found, along with mutations to *fliR/rcsA* and *nudE/yrfF* intergenic regions [26]. Each of these key mutated genes are hosted on different operons and their mutations either truncated their coding sequences or repressed their expression via mutations to their promoters and other non-coding features. All three genes belonged to the Rcs stress response system, whose activation by a perturbation (i.e., a *ptsHIcrr* knock out) in the starting strain was detrimental to population growth. The deactivation of the Rcs system through the mutations distributed across multiple operons was a key feature in the genotype which ultimately enabled the heightened aromatic amino acid levels. Though these types of mutation convergences can be manually identified with the prerequisite knowledge and detailed annotation, the growth in mutation datasets and the use of more complex organisms render their identification less likely.

It was observed that the addition of broader types of annotations to the multiscale annotation framework generally increases the number of key mutations found within ALE experiments (Fig. 3d). This is demonstrated by the increase in the median proportion of significantly convergent mutations (*P* < 0.05, permutation test, mutations to feature > 1) in ALE experiments with each broader annotation type. For example, 62% (median) of the mutated features annotated only with genomic features (i.e., coding and non-coding features) were found to be significant. When considering the COGs for these small features, 71% (median) of mutated features were involved in significant convergence.

#### Top-down functional analysis and mutated feature-to-condition associations for *E. coli* K-12 MG1655 growth selection on glycerol highlights changes to carbon catabolism and its repression

The methods of top-down functional analysis and mutated feature-to-condition association analysis contextualized mutations in an ALE experiment evolving *E. coli* K-12 MG1655 with some variants [27] on glycerol as a carbon source and resulted in an understanding of key mutations. The ALE experiment was previously executed with 30 independent replicates to test the effects of passage volume on endpoint adaptive phenotypes and represents a relatively large scale ALE experiment [23]. Besides passage volumes, the conditions used were similar to the earlier glycerol evolution of Herring et al. 2006 [28]. The replicate ALE endpoints were represented by a total of 51 samples (24 clonal, 27 population). Of the 51 total endpoints, 18 were represented with both clonal and population samples, while 6 endpoints were represented with one or more population samples, and 6 were represented with one or more clonal samples. Comprehensive whole-genome resequencing was performed for this work. Only mutations with a sample frequency of 50% or more were considered for population samples and those mutations that overlapped between endpoint samples were only considered once (see Methods). Collectively, 148 mutations were analyzed, representing a large ALE experiment mutation set for manual mutation functional analysis.

**Fig. 4:**
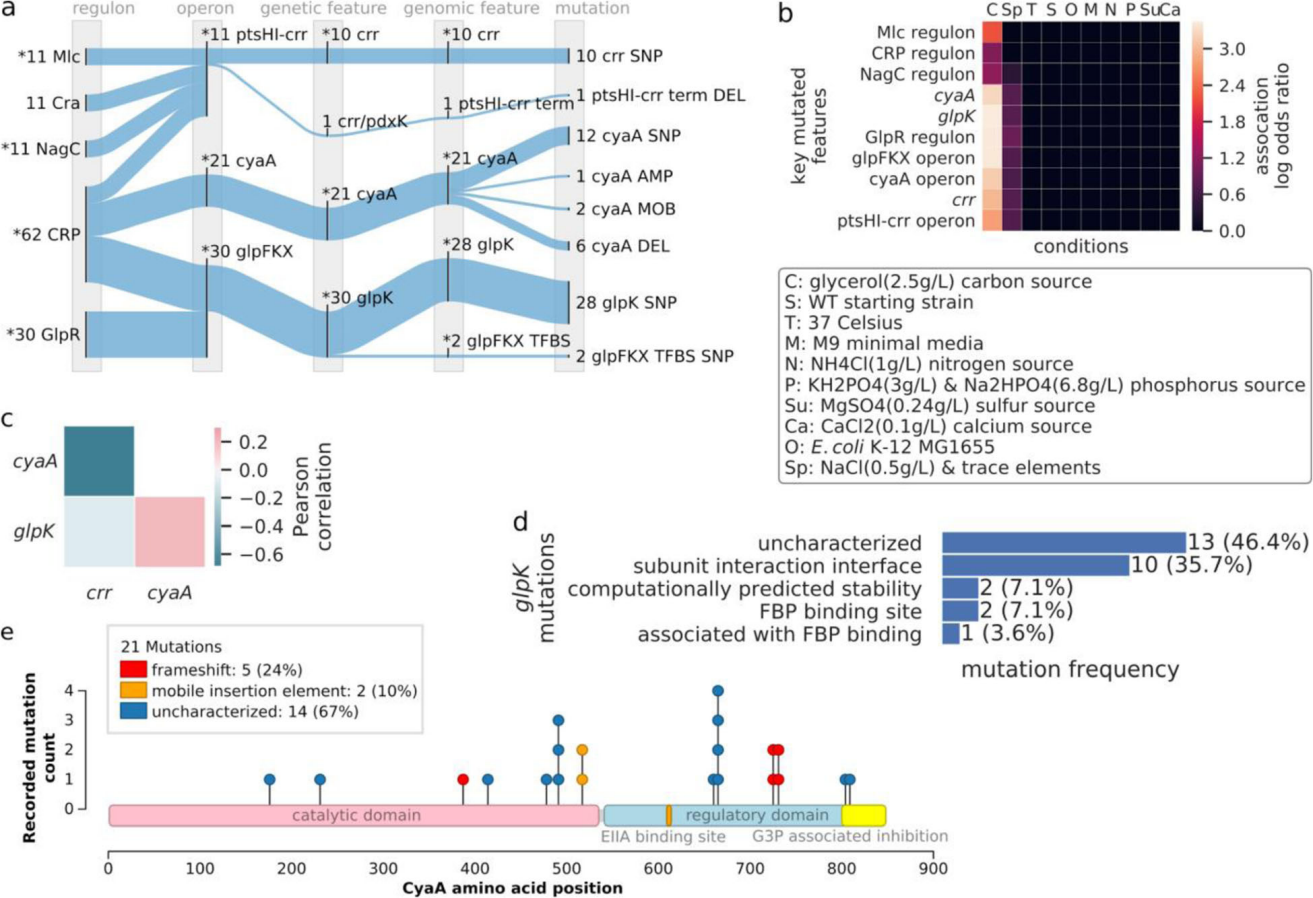
The relationships between mutated features regulated by CRP, their associations to glycerol as a carbon source, and the mutation effects on their coding sequences. **a** Mutated features that concurrently converge onto the CRP, Mlc, and GlpR regulons; an asterisk (*) denotes those features found to be mutated with a statistically significant amount relative to all mutations within the ALE experiment (P < 0.05, permutation test). The *glpK*, *crr*, and *cyaA* genes of interest all converge onto the CRP regulon. **b** A heatmap of the log odds ratios for features from Fig. 4a involved in significant mutation convergence (P < 0.05, permutation test, mutations to feature > 1) and their significantly associated conditions (*P* < 0.01, Fisher’s exact test). “att” is used to abbreviate “attenuator terminator”. **c** Heatmap of the Pearson correlation of mutation co-occurrence between *cyaA* and *crr*. **d** The frequency of characterizations to *glpK* mutations. **e** Mutation needle plot demonstrating the types of mutations in the amino acid sequence of the gene *cyaA*. The large light-blue domain is the *regulatory domain*, the small orange domain is the *EIIAGlc binding site*, and the yellow domain is the *G3P associated inhibition* region

The CRP regulon hosted 62 mutated features, the largest amount within this ALE experiment. The CRP regulon describes the functions associated with the dual regulator CRP and the cAMP receptor protein, where cAMP is known as the catabolite gene activator protein. CRP is known to regulate many functions, one of which is carbon catabolite repression (CCR) [29, 30] and represses the metabolism of carbon sources besides glucose. The *glpK, cyaA,* and *crr* genes were the three most often mutated in this experiment, hosting 28, 21, and 10 mutations, respectively, and are within the CRP regulon (Fig. 4a). The CRP regulon and genes were significantly converged upon by mutations (all *P* < 0.001, permutation test, mutations to feature > 1) and mutations to these genes were strongly associated with the selection pressure of glycerol as a carbon source (*P* < 0.001, Fisher’s exact test, Fig. 4b). In a similar study [28], the *glpK*, *crr*, and *cyaA* genes were also identified as being key to adaptation on glycerol as a carbon source. These associations, along with their convergence on the CRP regulon, suggest the mutations to *glpK*, *crr*, and *cyaA* as likely selected by the pressure to increase growth rates by rapidly utilizing glycerol as a carbon source.

The significant convergence of mutated features on functional annotations contextualized the targets of the *glpFKX* operon mutations. The significantly convergent *glpFKX* operon (*P* < 0.001, permutation test, mutations to feature > 1) hosted 30 instances of mutated features, corresponding to 28 coding SNPs in *glpK*, where two of these mutate a GlpR TFBS (located inside the coding sequence of *glpK*). These mutated features significantly enriched the GlpR regulon (*P* < 0.001, permutation test, mutations to feature > 1, Fig. 4a). The GlpR regulon represents the repression of glycerol transport and metabolism in the presence of glucose and the absence of glycerol or glycerol-3-phosphate for *E. coli* K-12 MG1655 [31]. The *glpR* genes of some *E. coli* K-12 MG1655 are pseudogenized. Similar to the U00096.2 *E. coli* K-12 MG1655 reference genome [32], the starting strain used in this experiment has a functional *glpR* [27]. The *glpFKX* operon mutations also significantly converged on the glycerolipid metabolism pathway (Supplemental Figure 1), which describes the chemical reactions involving any lipid with a glycerol backbone. The mutations to *glpK* may be working to increase the reaction rate of its product, glycerol kinase (GlpK), which catalyzes the phosphorylation of glycerol and is a rate-limiting enzyme in glycerol metabolism. GlpK is allosterically inhibited by fructose-1,6-bisphosphate (FBP) [33] and the *crr* gene product, Enzyme II A (IIAGlc) [34]. Two SNPs were found in an FBP binding site of GlpK, and 1 SNP substituted an amino acid that had been previously observed to abolish FBP regulation altogether (Supplemental Table 2). Ten SNPs targeted regions that are used in forming the GlpK oligomer and two SNPs were predicted to affect structural stability. GlpK is found to either form a tetramer or dimer, where FBP can inhibit the tetramer’s catalytic reaction. Mutations to the subunit interface regions that bias GlpK towards dimer formation, and therefore avoid FBP inhibition, have been seen in a similar glycerol evolution study [35]. The 2 SNPs that were predicted to affect structural stability may be accomplishing the same result. Overall, 53% of the mutations to *glpK* have effects that could disable inhibition by FBP (Fig. 4d).

Though there were approximately twice as many mutations in *cyaA* as there were to *crr*, these mutations may have accomplished similar adaptive effects. The presence of mutations to either of these targets was inversely correlated with the other (Fig. 4c), indicating antagonistic epistasis. Adenylate cyclase (AC), *cyaA*’s product, synthesizes cyclic adenosine monophosphate (cAMP). A cAMP molecule will bind with CRP and promote the transcription of CCR genes involved in secondary carbon source metabolism, including the *glpFKX* operon. Before cAMP can do so, this functionality of AC must be activated by binding with a phosphorylated IIAGlc. In the presence of phosphotransferase system (PTS) sugars such as glucose, IIAGlc will instead bind and inhibit GlpK. In the absence of PTS sugars, IIAGlc will become phosphorylated, bind, and activate AC’s ability to produce cAMP, ultimately reducing the effect of CCR [36].

A large proportion of the mutations to the *cyaA* gene had a disruptive effect. *cyaA* hosted a total of 21 unique mutations. The mutations that affect *cyaA* were of different types: SNPs, deletions, and mobile element insertions (MOB). One-third of the mutations to *cyaA*, the two MOBs and five frameshifting deletions, had disruptive effects on the coding sequence (Fig. 4e). AC is thought to have an N-terminal catalytic domain and C-terminal regulatory domain [37]. IIAGlc and glycerol-3-phosphate (G3P) are both associated with interactions on the regulatory domain, where phosphorylated IIAGlc is known to bind to amino acid 609 and activate cAMP production [36], and G3P is thought to lower AC’s cAMP production through a feature within the C-terminal’s final 48 amino acids [38]. The disruptive mutations to *cyaA* each affected the subset of AC’s features downstream of the mutation (Fig. 4e). The variety of features affected, including the features necessary for AC’s activity, provides evidence of the non-essentiality of AC in this evolution.

The evidence of potential mutation effects to *crr* also suggested the possible non-essentiality of IIAGlc in this evolution. The convergence of mutations to *crr* resulted in statistically significant enrichment of the Mlc and NagC regulons (Fig. 4a, Mlc *P* < 0.001, NagC *P* = 0.0007, permutation test, mutations to feature > 1), which both describe the regulation of the PTS system, a key contributor to the CCR system. All mutations to *crr* landed in the PTS EIIA type-1 domain, which hosts the IIAGlc phosphorylation site. 70% of the mutations to *crr* were predicted to have a structurally destabilizing change on their host structure (ΔΔGpred > 2, *crr* Supplemental Table 1). 90% of mutations to *crr* were predicted to have deleterious consequences to conserved regions (SIFT score > 0.05). Additional evidence towards the mutations having a disruptive effect on *crr* is the possible epistatic relationship between *cyaA* and *crr* mutations along with the mutations to *cyaA* having the clear potential to disrupt its functionality. This evidence suggested the potential non-essentiality of IIAGlc in this evolution.

This ALE experiment generated 148 mutations, an amount prohibitive to comprehensive manual functional analysis. The methods of top-down functional analysis and selection pressure associations generated valuable evidence that revealed a potential solution for increasing glycerol metabolism while maintaining the repression of metabolic systems for other secondary carbon sources. Of the many mutated regulons for this ALE experiment, three were emphasized by being both significantly converged upon by mutations (all *P* < 0.001, permutation test, mutations to feature > 1) and significantly associated with the designed selection pressure of growth on glycerol (all *P* < 0.001, Fisher’s exact test). The functions of these regulons contextualized their mutated genes, further informing on the biological systems potentially targeted by adaptation. Further investigation into the mutated genes informed on the possible beneficial mechanistic effects of their mutations. The mutations to *glpK* suggest the increase in its reaction rate through the disruption of an inhibition mechanism. The mutations to *cyaA* and *crr* suggest the disruption of cAMP synthesis, resulting in CCR maintenance in the presence of a carbon source that would normally dampen CCR. Such induction of CCR with mutations resulting from an experimental evolution on glycerol has been previously observed [35]. These results serve to promote the value of the evidence generated by the methods of this work in finding and understanding key mutations for ALE experiments with many mutations. There remains more mutations in this ALE experiment, though the mutations to *glpK, cyaA*, and *crr* had the strongest and most interpretable signals of adaptation.

#### Top-down functional analysis and mutated feature-to-condition associations for an *E. coli* K-12 MG1655 derived strain and growth selection for L-serine tolerance highlights changes to the glycine cleavage and transport system and global regulators

The methods of top-down functional analysis and mutated feature-to-condition association analysis contextualized mutations in an ALE experiment tolerizing a genetically engineered *E. coli* K-12 MG1655 strain to L-serine [24], resulting in previously unreported key mutations. This tolerization ALE experiment involved three independent replicate evolution experiments on a strain of *E. coli* K-12 MG1655 that had been genetically engineered to remove internal L-serine degradation pathways with the intention of higher L-serine production for industrial applications. ALE was necessary to tolerize the strain to the toxicity of high L-serine concentrations. The replicate endpoints were each represented by 2 clonal isolates. Comprehensive whole genome resequencing for the study was performed for this work. Mutations that overlapped between endpoint samples were only considered once (see Methods). Collectively, 27 mutations unique to endpoints were analyzed for this ALE study. The original Mundhada et al. study [24] revealed mutations to *thrA, lrp*, *rho, argP, pykF,* and *eno* contributed to L-serine tolerance and fitness in minimal-media.

**Fig. 5:**
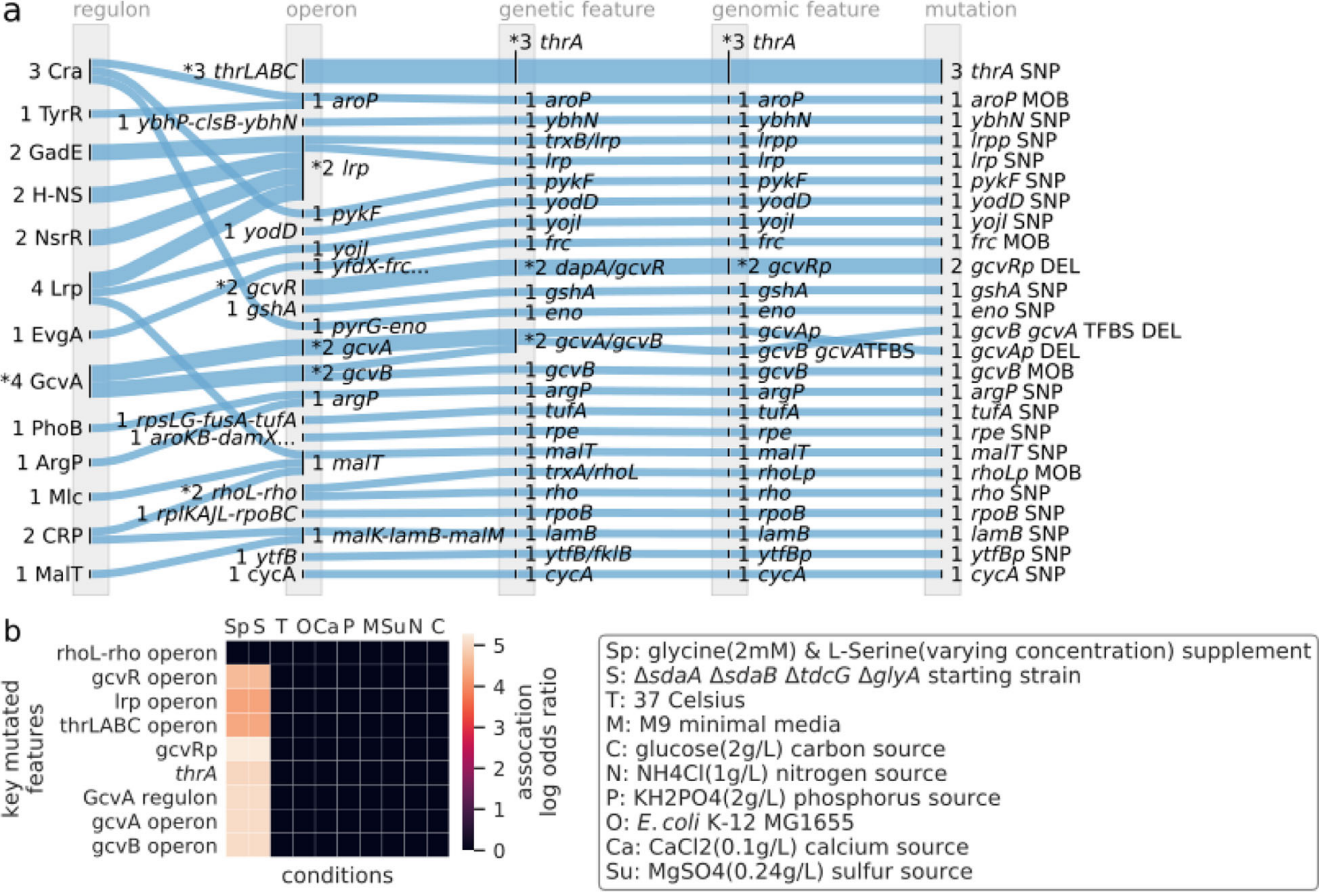
The convergence of mutated features and their associations for an L-serine tolerization ALE. **a** The convergence of mutated features from the Mundhada et al. 2017 ALE experiment onto regulons. **b** A heatmap of the odds ratios for significantly mutated features from the Mundhada et al. 2017 ALE experiment and their significantly associated conditions. Significantly mutated regulon and operons (*P* < 0.05, permutation test, mutations to feature > 1) were found to be significantly associated (*P* < 0.01, Fisher’s exact test) with the conditions of L-serine tolerance and the specific *E. coli* K-12 MG1655 starting strain. These features were associated with both conditions in equal magnitude and significance

The GcvA regulon, representing the glycine cleavage function, was significantly converged upon (*P* = 0.0013, permutation test, mutations to feature > 1) by mutated features (Fig. 5a). The starting strain for this ALE experiment was auxotrophic for glycine, therefore glycine was added to the media. Mutations that could offset a glycine deficiency would be beneficial. The GcvA regulon was also found to be significantly associated with the starting strain mutations and L-serine tolerance (Fig. 5b).

The operon level of annotations contained the largest amount of significantly convergent features for this ALE experiment, where all significantly mutated operons (gvcA *P* = 0.0046, remainder *P* < 0.001, permutation test, mutations to feature > 1) except *rhoL-rho* were significantly associated (*P* < 0.001, Fisher’s exact test) with L-serine tolerance and starting strain mutations (Fig. 5b). Due to hosting the largest amount of significantly convergent features, the operon level of annotations was the most revealing in the adaptive changes for this ALE experiment. By observing the mutations to operons, we can better understand non-coding mutations and their potential effect on gene products and the systems they contribute to. Regulatory genes for the glycine cleavage system host mutations in the regulatory features of their transcription units. The *gcvA* operon contains mutations in its promoter and a repressive GcvA TFBS (Fig. 5a). The *gcvA* gene encodes for a transcriptional dual regulator for the glycine cleavage system operon *gcvTHP* [39, 40]. Downregulating *gcvA*’s transcription could inhibit its activation of *gcvTHP*’s transcription. GcvA can also form a glycine cleavage repression complex with GcvR [40]. The *gcvR* gene’s promoter (*gcvRp*) experienced a 1 bp DEL in two endpoints (Fig. 5a). Upregulating *gcvR*’s transcription could have the effect of further repressing the gcvTHP operon coding for glycine cleavage. GcvR is additionally inhibited by glycine [41]. Mutations to *gcvA* and *gcvR* regulatory features may simply be removing their presence through the alteration of their promoters, leaving the glycine cleavage system operon with an unstimulated transcription rate. The *gcvB* operon, which plays a role in the glycine transport system and is co-regulated with the glycine cleavage system through GcvA, hosted mutations to two different features. The *gcvB* gene of one endpoint hosted a mobile element insertion (i.e., MOB), where another endpoint hosted a 1 bp DEL in an activating GcvA TFBS (Fig. 5a). The *gcvB* gene encodes a small regulatory RNA that acts as a repressor of *cycA* [42], which also hosts a SNP, and functions as a symporter of glycine, D-serine, and D-alanine [43]. Disruption of *gcvB* may increase the uptake of glycine by disabling the repression of CycA. The original study recognized the mutations to the *gcvB* operon as potentially beneficial. The emphasis on the glycine cleavage system due to the significant convergence of the GcvA regulon by mutations suggests mutations to the *gcvA* and *gcvR* operons as additionally being key.

The operons for global regulators *lrp* and *rho* were both significantly enriched by the convergence of their mutated coding sequences and promoters (*P* < 0.001, permutation test, mutations to feature > 1). *lrp* is a global regulator for the leucine response system and has experimental evidence demonstrating its participation in L-serine tolerance [24]. The roles of these global regulators in this adaptation remains unknown due to their large network of interactions, though the *lrp* and *rho* coding sequence mutations were found to contribute the most fitness among all endpoint mutations of this experiment [24].

This ALE experiment generated a variety of mutations to both coding and non-coding regions, where mutations to non-coding features not considered in the original study provided evidence for new key mutations. The methods of top-down functional analysis and selection pressure associations generated valuable evidence that revealed a potential optimization for the strain’s glycine auxotrophy. The richer annotations for non-coding features on the genome revealed the high frequency of regulatory features targeted by ALE mutations and facilitated the discovery of previously unrecognized mutated regulatory features. Significant enrichment of features on the multiscale annotation framework emphasized the systems in which the ALE mutations affected, informing on the normally more elusive non-coding mutations. This emphasis on higher-level features revealed previously unconsidered changes to the glycine cleavage system that could benefit the host strain. These results serve to promote the value of the evidence generated by the methods of this work in finding and understanding key mutations.

## Discussion

To better identify and understand causal mutations from ALE experiments, this study developed a multiscale genome annotation framework and applied a statistical enrichment method for mutated features across annotation scales. Using this framework, it was found that (1) ALE mutations target a variety of regulatory features including promoters, attenuator-terminators, and ribosomal binding sites, (2) mutated non-coding regulatory features were often involved in significant convergence events, and (3) the method of bottom-up convergence from small to large features on the multiscale annotation framework found more key mutations than when considering only genes and intergenic regions for mutation convergence. The convergence of mutated features onto broader functional annotations additionally enabled a top-down approach to mutation functional analysis, where one can first consider biological functions hosting mutations before investigating the numerous smaller mutated features. Further, we computed statistical associations between a large set of experimental evolution conditions and mutated features. These associations provided evidence towards clarifying the selection pressures for mutated features among the multiple conditions describing each ALE experiment. Taken together, the results presented here have several implications.

First, the identification of key mutations was enhanced by seeking mutations involved in statistically significant convergence events across multiple scales of genome annotation. By including regulatory feature annotations alongside genes, a larger proportion of an experiment’s mutations were placed in features with known functions, which were subsequently connected to broader annotation types such as operons and functional annotations. Being able to connect more mutations to known features resulted in more convergence events across the annotation framework. Additionally, the application of a statistical enrichment test can be used to prioritize convergence events in the followup work of mutation functional analysis. This will become more important as ALE experiments increase in scale. All mutated regulatory feature types were involved in statistically significant convergence events, and except for promoters, had a higher median proportion of their mutations involved in these events than mutated genes. These results suggest that mutations to non-coding regulatory features should be considered as important as coding features when searching for key mutations, though standard genome annotations often lack non-coding regulatory features. When only considering mutations from significantly converged upon features, the amount of significant or key mutations on larger-scale annotation types (operons, pathways, regulons, COGs) remains larger than those on smaller annotation types (e.g., genes, promoters). The convergence of a variety of small mutated features onto broader annotations also serves to maximize the amount of evidence used in identifying the overall changes of an adaptive genotype. In its application, this method identified new regulatory key mutations for a published ALE study on L-serine tolerance [24], demonstrating the value of richer annotations and convergence across multiple scales of annotation. Additionally, significant convergence involving the most beneficial mutations to the L-serine tolerance evolution, those to the coding sequences and regulatory features of the *lrp* and *rho* genes, was made explicit with the inclusion of regulatory features in mutation annotations. These approaches can therefore lessen the challenge of finding key mutations by providing enhancements to traditional methods.

Second, the functional analysis of key mutations was enhanced by considering the functions of the features upon which mutations converged and the conditions that mutated features were associated with. We found that a top-down mutation functional analysis proved valuable in contextualizing key mutations according to the high-level functional annotations to which they were connected and shared with other key mutations. The three most frequently mutated genes of strains from the ALE experiment on glycerol [23] all converged on the CRP regulon, revealing the functional proximity of their products through their participation in the CCR system. This convergence served to inform how mutations to these genes could have manipulated the CCR system to enable only the catabolism of glycerol and maintain the repression of the remaining secondary carbon source catabolism systems. The new key mutations proposed for the L-serine ALE study [24] were additionally enhanced by this top-down functional analysis approach with the significant convergence of the GcvA regulon, describing their involvement in the glycine cleavage system. The starting strain for this experimental evolution was auxotrophic for glycine and had to have it provided as a supplement. This convergence informed how these mutated features could alleviate the strain’s glycine auxotrophy by either repressing the glycine cleavage system to conserve glycine or increasing the uptake of glycine. Similarly, the association of mutated features to conditions can deconvolute the conditions selecting for mutations. The three most prominent key mutated genomic features of the glycerol ALE were primarily associated with the condition of glycerol as a carbon source, further strengthening the functional analysis’ focus on glycerol metabolism. The key mutations of the L-serine ALE with genomic features mutated more than once were likewise uniquely associated with the conditions of the starting strain and high concentrations of L-serine. Mutated feature-to-condition association is valuable to mutation interpretation in that researchers can focus their efforts on how these mutations enable adaptation to the significantly associated conditions of interest. These methods can lessen the challenge of understanding key mutations by providing enhancements to traditional methods.

The methods presented here demonstrate the value in leveraging the diverse available resources describing ALE variants, but are not without limitations. The procedure for finding the statistical significance of mutation convergence assumes that each nucleotide on the genome has the same probability of being spontaneously mutated, leading to features having the probability of being mutated proportional to their length in nucleotides. Nucleotides can have varying mutation rates; for example, wild type *E. coli* indel mutation rates have been observed to be higher in mononucleotide runs of 4 or more [18]. Parameterizing different genomic features or nucleotide locations with better representative mutation rates would enable increased accuracy in the significance measurements of mutation convergence events. There additionally exist numerous annotation frameworks currently not integrated, such as structural annotations [44, 45], gene product complexes [46], and gene ontologies [47, 48]. Their inclusion would increase the coverage of biological domains for the methods this work. The abundance of annotation frameworks is often limited to model organisms such as *E. coli* K-12 MG1655. To use these methods with other organisms would require knowledge bases that describe the genes and regulatory architecture of those organisms’ genome, along with the biological functions that the genes contribute to. Evolution experiments often also include midpoint or intermediate samples. The results of this work only included endpoint samples, as not all experiments had midpoints, to enable a uniform analysis method across all experiments. The inclusion of midpoint samples could provide further evidence of mutation convergence through the dynamics of clonal interference, as well as the opportunity for mutation time series analysis. Correlations between mutated features proved useful for interpreting the relationship between the mutated *cyaA* and *crr* genes from the glycerol carbon source ALE case study. Applying the same correlation method on a larger scale mutated feature set tends to generate less interpretable results, though statistical evidence of relationships between mutated features would still be valuable. More sophisticated methods for establishing these relationships within large-scale sets of mutated features are necessary to extract meaningful interpretations. Finally, the key mutations found in this work were derived from sets of mutations of a scale that still can be managed manually, albeit with great effort. As ALE datasets grow and automation-enabled studies increase in overall sample number, this work’s methods will become necessary to comprehensively consider and understand an ALE experiment’s mutations.

## Conclusion

Here, we have reported on data-driven approaches to better find and understand ALE key mutations. We demonstrated how a multiscale genome annotation framework and statistical association methods can better identify and characterize adaptive mutations generated through controlled evolution experiments. We anticipate that this workflow will be leveraged in the future to provide deeper insights into the vast amounts of -omics data that are generated for targeted microorganisms amenable to ALE and provide the blueprints for similar data-driven annotation frameworks.

## Methods

### ALE experiments

This work focuses on ALE experiments with a starting strain of *E. coli* K-12 MG1655 or derivative thereof, due to the availability of experimental data and genome annotations. Thirty five ALE experiments, each using a unique set of conditions and selection pressures, were exported from ALEdb [16] for this work [8, 21, 23, 24, 26, 49–57]. These ALE experiments consist of 208 evolutions and 2641 mutations. Mutations in population samples with a frequency below 50% were filtered out to instead focus on mutations that demonstrate dominant selection within a sample. When inspecting experiments for key mutations, endpoint samples containing known hypermutator strains were discarded in an effort to focus on less complex genotypes.

### ALE experiment mutation organization

Only mutations from endpoint flask samples (i.e., the flask that contained the final sequenced sample) were considered for the ALE experiments used in this work. Both clonal and population samples were included in this work. If the same mutation was available in more than one sample from an endpoint flask, the instance of the mutation with the highest sample frequency was chosen to represent all instances of the mutation from the flask.

### ALE experiment conditions

The ALE experiment conditions metadata used in the associations was gathered from the metadata reports available from ALEdb [16] for each ALE experiment considered in this work.

### Software for analysis and figure generation

#### Quantitative plots

Unless otherwise stated, figure plots were generated using *Matplotlib* version 3.0.3 [58] and *seaborn* version 0.9 Python software packages [59].

#### Flow diagrams

The mutation flow diagrams of Figs. 1c, 3a, 4a, 5a and Supplemental Fig. 1 were generated using the *Floweaver* Python software package [60].

#### Mutation needle plot diagram

The mutation needle plot of Fig. 3e was generated using the *muts-needle-plot* Javascript software package [61].

### Mutation calling

The *breseq* pipeline version 0.33.1 [62] was used to map the DNA-seq reads to an *E. coli* K-12 MG1655 reference genome. The reference genome was either the NCBI accession NC_000913 version 3 reference genome or a derivative based on this NCBI genome [63]. DNA-seq quality control was accomplished using the software AfterQC version 0.9.7 [64].

### Mutation effect prediction

The predicted disrupted effects of mutations to the genes in the glycerol evolution results were generated using the *mutfunc* web application [65].

### Software scripts

Software scripts and data essential to the publication is available through a github repository [66].

## Supplementary information

**Additional file 1:** Supplementary figures and tables.

## Data Availability

The datasets supporting the conclusions of this article are available in the github repository [66], ALEdb database [16], and within this article’s additional files.

## Abbreviations

ALE: Adaptive laboratory evolution
COGs: Cluster of orthologous groups
DAG: Directed acyclic graph
TU: Transcription unit
TFBS: Transcription factor binding site
RBS: Ribosomal binding site
SNP: Single nucleotide polymorphism mutation
DEL: Deletion mutation
INS: Insertion mutation
MOB: Mobile element insertion mutation
CCR: Carbon catabolite repression
FBP: fructose-1,6-bisphosphate
EIIA: Enzyme II A
IIAGlc: Enzyme IIA (Glc)
AC: Adenylate cyclase
cAMP: Cyclic adenosine monophosphate
PTS: Phosphotransferase system
ΔΔGpred: The difference between the free energy of unfolding the protein structure before and after a mutation
SIFT: Sorting intolerant from tolerant
