# Supplementary material for: Causal mutations from adaptive laboratory evolution are outlined by multiple scales of genome annotations and condition-specificity
Source: BMC Genomics. 2020 Jul 25;21:514. doi: 10.1186/s12864-020-06920-4 (PMC7382830; doi:10.1186/s12864-020-06920-4)
Supplement: Supplementary file 1 — Additional file 1: Supplementary figures and tables. [file 12864_2020_6920_MOESM1_ESM.docx]

# **Supplemental**

## **Supplemental mutation flow diagrams**


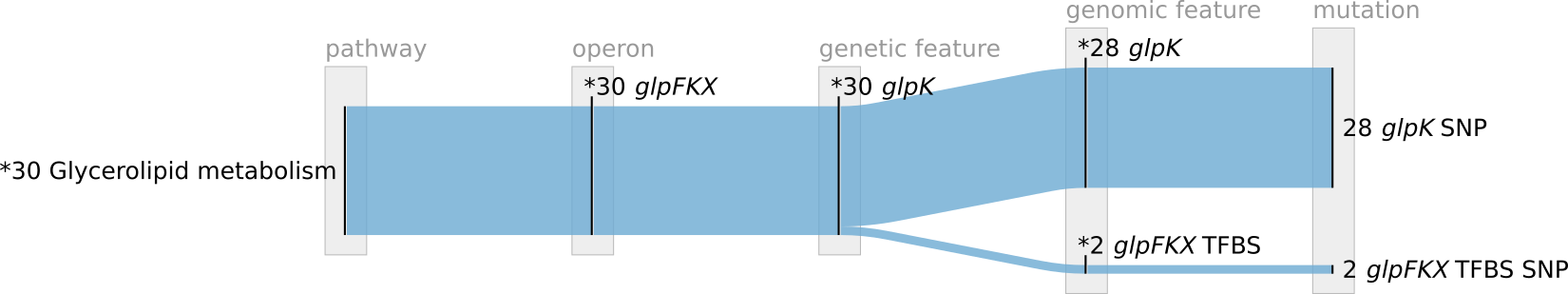


Supplemental Figure 1 The mutation convergence and significance of features associated with *glpK* and glycerol metabolism pathway.

| start AA position | stop AA position | mut type | details | ΔΔGpred (destabilizing) | SIFT score (deleterious) |
| --- | --- | --- | --- | --- | --- |
| 34 | 34 | SNP | I34T | 3.47091 | 0.0074979 |
| 39 | 39 | SNP | D39H | 2.49214 | 0 |
| 44 | 44 | SNP | E44V | NA | 0 |
| 48 | 48 | SNP | G48V | 18.6249 | 0 |
| 50 | 50 | SNP | G50S | 13.6354 | 0 |
| 72 | 72 | SNP | F72V | NA | 0 |
| 72 | 72 | SNP | F72V | NA | 0 |
| 93 | 93 | SNP | G93S | 7.2727 | 0 |
| 138 | 138 | SNP | P138Q | 3.46287 | 0 |
| 138 | 138 | SNP | P138Q | 3.46287 | 0 |

Supplemental Table 1 Table of *crr* mutation details.

| AA position | AA change | summary |
| --- | --- | --- |
| 17 | S17A | uncharacterized |
| 34 | R34H | uncharacterized |
| 34 | R34S | uncharacterized |
| 54 | W54L | uncharacterized |
| 55 | A55T | uncharacterized |
| 56 | T56A | uncharacterized |
| 59 | S59Y | subunit interaction interface [(1)](https://paperpile.com/c/Z4cYRv/JJ0y) |
| 59 | S59Y | subunit interaction interface [(1)](https://paperpile.com/c/Z4cYRv/JJ0y) |
| 59 | S59Y | subunit interaction interface [(1)](https://paperpile.com/c/Z4cYRv/JJ0y) |
| 59 | S59Y | subunit interaction interface [(1)](https://paperpile.com/c/Z4cYRv/JJ0y) |
| 66 | A66S | associated to FBP binding [(2)](https://paperpile.com/c/Z4cYRv/uBZ5Y) |
| 73 | D73V | subunit interaction interface [(3)](https://paperpile.com/c/Z4cYRv/fu3Bh) |
| 73 | D73V | subunit interaction interface [(3)](https://paperpile.com/c/Z4cYRv/fu3Bh) |
| 73 | D73V | subunit interaction interface [(3)](https://paperpile.com/c/Z4cYRv/fu3Bh) |
| 73 | D73V | subunit interaction interface [(3)](https://paperpile.com/c/Z4cYRv/fu3Bh) |
| 73 | D73A | subunit interaction interface [(3)](https://paperpile.com/c/Z4cYRv/fu3Bh) |
| 73 | D73A | subunit interaction interface [(3)](https://paperpile.com/c/Z4cYRv/fu3Bh) |
| 96 | K96E | uncharacterized |
| 96 | K96Q | uncharacterized |
| 96 | K96Q | uncharacterized |
| 96 | K96Q | uncharacterized |
| 97 | P97H | Computationally predicted stability [(4)](https://paperpile.com/c/Z4cYRv/mfiMs) |
| 173 | K173T | Computationally predicted stability [(4)](https://paperpile.com/c/Z4cYRv/mfiMs) |
| 229 | N229H | uncharacterized |
| 229 | N229K | uncharacterized |
| 236 | T236K | FBP binding site [(5)](https://paperpile.com/c/Z4cYRv/RN0V5) |
| 236 | T236K | FBP binding site [(5)](https://paperpile.com/c/Z4cYRv/RN0V5) |
| 441 | A441V | uncharacterized |

Supplemental Table 2 Table of *glpK* SNP details.

**References**

1. [Bystrom CE, Pettigrew DW, Branchaud BP, O’Brien P, Remington SJ. Crystal structures of Escherichia coli glycerol kinase variant S58-->W in complex with nonhydrolyzable ATP analogues reveal a putative active conformation of the enzyme as a result of domain motion. Biochemistry. 1999 Mar 23;38(12):3508–18.](http://paperpile.com/b/Z4cYRv/JJ0y)

2. [Feese MD, Faber HR, Bystrom CE, Pettigrew DW, Remington SJ. Glycerol kinase from Escherichia coli and an Ala65→Thr mutant: the crystal structures reveal conformational changes with implications for allosteric regulation. Structure. 1998 Nov 15;6(11):1407–18.](http://paperpile.com/b/Z4cYRv/uBZ5Y)

3. [Herring CD, Raghunathan A, Honisch C, Patel T, Applebee MK, Joyce AR, et al. Comparative genome sequencing of Escherichia coli allows observation of bacterial evolution on a laboratory timescale. Nat Genet. 2006 Dec;38(12):1406–12.](http://paperpile.com/b/Z4cYRv/fu3Bh)

4. [Wagih O, Galardini M, Busby BP, Memon D, Typas A, Beltrao P. A resource of variant effect predictions of single nucleotide variants in model organisms. Mol Syst Biol. 2018 Dec 20;14(12):e8430.](http://paperpile.com/b/Z4cYRv/mfiMs)

5. [UniProt Consortium T. UniProt: the universal protein knowledgebase. Nucleic Acids Res. 2018 Mar 16;46(5):2699.](http://paperpile.com/b/Z4cYRv/RN0V5)
